# Drinking Habits and Physical Activity Interact and Attenuate Obesity Predisposition of *TMEM18* Polymorphisms Carriers

**DOI:** 10.3390/nu15020266

**Published:** 2023-01-04

**Authors:** Danyel Chermon, Ruth Birk

**Affiliations:** Nutrition Department, Health Science Faculty, Ariel University, Ariel 40700, Israel

**Keywords:** Transmembrane protein 18, single nucleotide polymorphisms, obesity, lifestyle, physical activity

## Abstract

The transmembrane protein 18 (*TMEM18*) gene plays a central and peripheral role in weight regulation. *TMEM18* genetic polymorphisms have been identified as an important risk factor for obesity, depending on ethnic population and age. This research aimed to study the association of common *TMEM18* polymorphisms with obesity and their interactions with modifiable factors, namely drinking habits (sugar-sweetened beverages (SSBs), flavored water and wine) and physical activity (PA) in the Israeli population. Adults (n = 3089) were analyzed for common *TMEM18* polymorphisms and lifestyle and nutrition habits were obtained from questionnaires using adjusted (age, sex) binary logistic regression models. *TMEM18* rs939583 and rs1879523 were significantly associated with increased obesity risk (OR = 1.35, 95% CI (1.17–1.57) and OR = 1.66, 95% CI (1.29–2.15), respectively). *TMEM18* rs939583 interacted with consumption of 1–3 weekly glasses of wine and PA to attenuate obesity risk (OR = 0.82 95% CI (0.74–0.9; *p* < 0.001) and OR = 0.74 95% CI (0.68–0.8), respectively), while physical inactivity, SSBs and flavored water consumption significantly enhanced obesity risk (OR = 1.54 95% CI (1.41–1.67), OR = 1.31 95% CI (1.14–1.51) and OR = 1.35 95% CI (1.13–1.62), respectively). PA duration was significantly associated with a lower BMI for rs939583 risk carriers, with a PA cutoff of >30 min/week (*p* = 0.005) and >90 min/week (*p* = 0.01). Common *TMEM18* SNPs were significantly linked with adult obesity risk and interacted with modifiable lifestyle factors.

## 1. Introduction

Obesity is a significant public health burden growing at a distressing rate [1]. Obesity is a major risk factor for the augmentation of related chronic diseases, such as cardiovascular diseases, type 2 diabetes mellitus (T2DM), hypertension, osteoarthritis, and several types of cancer, resulting in a decreased quality of life and longevity [2]. The etiology of obesity is diversified by genetic and environmental factors, where the latter has been reported to account for 40–70% of the population’s body mass index (BMI) variation [3]. Several to hundreds of inherited genetic common variants have been found to promote polygenic obesity development [4]. In parallel, the obesogenic environment is characterized by; excessive consumption of energy, sugar, fat, and processed foods, over and fast eating, and binge eating patterns, alongside a sedentary lifestyle expressed by inactivity and excessive screen time and sleeplessness, further favoring the obesity prevalence. This obesogenic environment promotes the excess intake of high and empty calories in parallel to reduced energy expenditure due to a lack of physical activity, which leads to an unbalanced caloric state, favoring weight gain. Furthermore, inadequate sleep can affect metabolism and circadian hormone levels, leading to weight gain [5,6]. With an elevated exposure to an obesity-promoting environment, obesity is primarily expressed in genetically predisposed obesity carriers [7].

The transmembrane protein 18 (*TMEM18*) gene, located on chromosome 2, is a protein-coding gene. *TMEM18* protein is highly expressed in the central nervous system, including brain regions such as the hypothalamus, the region in the brain regulating eating behavior [8]. *TMEM18* has been reported to be involved in weight regulation through central and peripheral routes, including; energy balance, central appetite control, and adipogenesis [9,10,11]. The *TMEM18* locus has been identified and confirmed by several subsequent genome-wide association studies (GWASs) for single nucleotide polymorphisms (SNPs) associated with obesity [8,12,13] and BMI [14]. The *TMEM18* obesity gene is highly conserved in different species that diverged from the human lineage [15]. However, differences in the *TMEM18* locus and obesity risk appear to exist between ethnic populations and age groups [10].

Clearly, association between obesity and *TMEM18* polymorphisms are needed to be confirmed to determine their precise effect in diverse populations. Furthermore, *TMEM18* locus obesity associated polymorphisms have been scarcely studied in relation to the interaction with modifiable environmental factors. Thus, we analyzed common *TMEM18* SNPs associated with obesity and actionable lifestyle factors in the Israeli population.

## 2. Materials and Methods

### 2.1. Participants

Israeli adults (n = 3089) with a mean age of 55.21 ± 14.31 were genotyped and asked to complete an online lifestyle questionnaire between 21.12.2021–1.10.2022. The analysis in this work was based on information listed in the Israeli registry database (#700068969) of Lev Hai Genetics LTD—MyGenes. The genetic database was anonymous. Ethical approval for this study was obtained from the Ethics (Helsinki) Committee of Ariel University (#AU-HEA-RB-20220214). Exclusion criteria included: <18 years age, occurrence of a genetic disorder, or missing genetic or anthropometric indices value (n = 61).

All study participants completed an online questionnaire in order to collect data on drinking habits and PA. PA habits questions, included: “Are you physically active” (optional answers: yes; no); “How many days a week do you engage physical activity” (answer: physically active days); “What is the duration of each physical activity you engage” (possible answers: 30 min; 60 min; more than 60 min). Consumption definition of SSBs and flavored water were; drinking ≥ 1 serving (12 fluid ounces)/daily of SSB or flavored water and the definition of regular wine consumption was 1–3 glasses (1 drink = 5 ounces)/week.

Anthropometric indices (weight and height) were self-reported. Weight was measured in kilograms and height was measured in centimeters. BMI was calculated as the ratio of weight/(height)^2^ (kg/m^2^). Subjects with a BMI ≥ 30 were classified as obese and subjects with BMI < 30 were classified as non-obese in accordance with the BMI cutoff points [16].

### 2.2. Single Nuclotide Polymorphism (SNP) Selection and Hardy–Weinberg Equilibrium (HWE)

Selected SNPs have been shown to be significantly associated with obesity in previous studies and prioritized based on their minor allele frequency (MAF) (>0.01), in no less than two GWAS populations [17,18,19] and in the validated catalog of published GWASs [20]. We analyzed the following SNPs: rs939583, rs1879523, rs2867125, rs2903492, rs4854344, rs6548238, rs10189761, rs13021737, and rs7561317. Each SNP was tested for Hardy–Weinberg equilibrium (HWE) using one degree of freedom χ^2^ test. Common TMEM18 SNPs were all in HWE (Table S1).

### 2.3. Statistical Analyses

An a priori power analysis was performed using G*Power 3.1.9.7 software [21], to determine the study sample size of 503 subjects to observe the association between obesity and common SNPs across two groups (odds ratio (OR) = 1.5 and a power of 0.95, α = 0.05). Continuous descriptive characteristics of the study participants are presented as mean values ± standard deviation. Χ^2^ statistical test was used for the comparison of dichotomized characteristic variables between participants defined as obese and participants defined as non-obese. Continuous variables that were not normally distributed were analyzed using the Mann–Whitney test for independence. We used binary logistic regression to elucidate the SNPs effect as predictors on the relative likelihood of being defined as obese for the following accepted genetic models; allelic, recessive, dominant, additive, and co-dominant. Regressions were adjusted for probable confounders (e.g., gender, age, and T2DM). To determine the OR of the common TMEM18 SNP obesity risk due to gene–environment interactions, we used logistic regression with interactions performed by a constructed model which included the independent variables: age, gender, T2DM, SSB consumption, flavored water consumption, wine consumption, and PA × genotype. We performed binary logistic regression to determine the effect of each modifiable environmental factor (e.g., drinking habits and PA) on SNPs obesity risk by a likelihood ratio test. We created the PA duration variable by summing the total physical activities duration per week for each participant. We compared each group to the reference group (inactive participants) and the previous group using a Mann–Whitney non-parametric test. Statistical analyses were performed using SPSS 29.0 for Windows (SPSS Inc., Chicago, IL, USA).

## 3. Results

### 3.1. Participants Characteristics

Adult participants (n = 3089) with a mean age of 55.21 ± 14.31, out of which 66.3% were female, were included in this cross-sectional study. Population characteristics are shown in Table 1. Gender, weight and BMI were significantly different between the obese and non-obese participants (*p* < 0.001, <0.0001 and *p* < 0.001, respectively). A significant difference was found between the obese and non-obese subjects for drinking habits and being active, in the following characteristics; the consumption of SSBs (≥1 serving/day), consumption of flavored water (≥1 serving/day), weekly wine drinking and PA. In Particular, obese-defined subjects were significantly less engaged in PA, consumed higher amounts of SSBs and flavored water and were less likely to regularly drink wine compared to non-obese subjects (*p* < 0.001, *p* < 0.001, *p* = 0.02 and *p* < 0.001, respectively). There was no difference in age, height or smoking status between the obese and non-obese subjects.

### 3.2. TMEM18 SNPs Associated with Obesity Risk

We analyzed *TMEM18* rs939583, rs1879523 and surrogate SNPs previously reported to be associated with obesity across five genetic models: allelic, dominant, recessive, additive and codominant. All SNPs showed a significant association with the elevated risk of obesity with ORs ranging between 1.19–1.73. Common *TMEM18* SNPs presented a high linkage disequilibrium (LD) (r2 = ~1) corresponding to the CEU population. We focused on *TMEM18* rs939583 to represent all other analyzed SNPs. In addition, we chose to analyze rs1879523 for its different genetic distribution compared to the other analyzed SNPs. Yet, rs1879523 distribution corresponded to other populations’ *TMEM18* SNP genotype distributions according to the dbSNP reference SNP (rs) report. The SNP genotype distributions and ORs accepted genetic models, adjusted for potential confounders are shown in Table 2.

Of the study subjects analyzed for *TMEM18* rs939583, 5.4% were homozygous to the reference (REF) allele (CC), 34.3% were heterozygous (TC), and 60.2% were homozygous to the polymorphic alternation (ALT) allele (TT). The frequency of the rs1879523 genotypes was; 47.2% for homozygous REF allele (AA), 34.4% heterozygous (AT) and 9.4% homozygous to the ALT allele (TT). A minor allele frequency (MAF) of 77.35% and 31.1% for rs939583 and rs1879523 were slightly lower compared to MAF in the European population according to the NIH dbSNP report (84.23% and 31.7%, respectively) (Table S1). A mean increase of 0.91 and 0.95 BMI units were found for rs939583 ALT homozygotes (TT) compared to the mean BMI of heterozygotes carriers (*p* < 0.001) and REF homozygotes (CC) (*p* = 0.02), respectively. Homozygotes for the rs1879523 T allele had a mean of 1.23 BMI unit increase compared to heterozygotes (*p* < 0.005) and a mean 1.78 BMI unit increase compared to the A allele homozygotes (*p* = 0.02). For the allelic model, an elevated obesity risk was found for the rs939583 T allele carriers compared to the C allele carriers (OR = 1.27 95% CI 1.27–1.43; *p* = 0.00004), and for the rs1879523 A allele carriers compared to the T allele carriers (OR = 1.22 95% CI 1.09–1.36, *p* = 0.0002).

### 3.3. TMEM18 rs939583 and rs1879523 Interaction with Actionable Lifestyle Factors

Gene–environmental interactions were found for rs939583 and rs1879523 with actionable lifestyle variables; physical activity (PA) and drinking habits including; sugar-sweetened beverages (SSBs), flavored water and wine consumption. Being physically active at least 30 min/week and consuming wine in moderation (1–3 glasses/week) modulated and significantly attenuated the obesity risk of rs939583 and rs1879523 carriers. Whereas consuming ≥ 1 glass a day of SSBs and ≥1 glass a day of flavored water significantly elevated the obesity risk predisposition of rs939583 and rs1879523 carriers (Table 3).

#### 3.3.1. PA

Being physically active was found to be a significant protective actionable factor against obesity risk compared to being inactive in the rs939583 TT variant carriers. Physical activity duration was associated with BMI reduction with a consistently lower BMI as the activity duration increased. Significant BMI differences were found for two PA duration cutoff points; between physically inactive participants compared to those who were active a total of 30 min/week (*p* = 0.005) and between those who were active less than 90 min/week compared to those who were active >90 min/week (*p* = 0.01) (Table 4).

#### 3.3.2. Wine Consumption

As shown in Table 5, consuming 1–3 glasses of wine per week was found to have a protective effect against obesity risk for *TMEM18* rs939583 ALT allele carriers. Where *TMEM18* rs939583 ALT allele heterozygotes and homozygotes carriers who consume wine in moderation significantly reduced their obesity risk by 40 and 35%, respectively, compared to ALT allele heterozygotes and homozygotes carriers who did not consume any wine.

#### 3.3.3. SSBs

Consuming ≥1 glass per day of SSBs significantly elevated the risk of obesity for rs939583 ALT allele heterozygotes and homozygotes carriers compared to their genotype counterparts who did not consume any SSBs (OR = 1.78 95% CI 1.2–2.63; *p* = 0.004 and OR = 1.47 95% CI 1.08–2.04; *p* = 0.01, respectively) (Table 5).

#### 3.3.4. Flavored Water Consumption

rs939583 ALT allele homozygotes (TT) who consumed ≥1 glass per day of flavored water had a 2.14-fold elevated obesity risk in comparison to their genetic counterparts who did not consume flavored water. Among the participants who regularly consumed ≥1 glass per day of flavored water, ALT homozygotes (TT) carriers had a 2.69- and 6.14-fold higher obesity risk compared to ALT allele heterozygotes carriers and REF allele homozygotes, respectively (Table 6).

## 4. Discussion

We have shown that common *TMEM18* locus SNPs, rs939583 and rs1879523, are significantly associated with an elevated obesity risk in the adult Israeli population. The *TMEM18* gene is an obesity-associated gene involved in the regulation of the hypothalamic pathways that regulate appetite and body weight. It is believed to play a key role in the central control of body weight, as it is involved in signaling pathways that control energy balance and metabolism. Earlier studies have demonstrated the association of common *TMEM18* SNPs with a higher obesity risk in childhood, partly due to the down-regulation of *TMEM18* expression in adipocytes leading to adipocyte disfunction. Regarding adults, studies on the common *TMEM18* SNPs associated with obesity have been inconsistent across different populations, pointing to possible population-related associations [9,10,14].

Our findings show that carriers of the *TMEM18* rs939583 and rs1879523 SNPs have a significantly elevated obesity risk (OR = 1.21 95% CI 1.06–1.39; *p* = 0.002) and for the rs939583 C allele, which has a slightly lower risk (OR = 1.32 [1.10–1.59]) found for Europeans descendants [9]. *TMEM18* rs939583 significantly interacted with actionable lifestyle factors, namely, PA and drinking habits (SSBs, flavored water and wine). PA significantly attenuated the *TMEM18* rs939583 ALT allele homozygotes (TT) obesity risk compared to the inactive ALT allele homozygotes carriers. Furthermore, our findings showed that PA duration was positively associated with consistent BMI reduction, with two significant PA duration cutoffs of >30 min/week and >90 min/week. Thus, indicating that the attenuation of the obesity risk and a reduction in BMI of the *TMEM18* rs939583 homozygotes (TT) carriers could be achieved through engagement in PA for at least (minimum) 30 min/week with further BMI reduction when engaging in PA for >90 min/week. It is well known that inactivity plays a key role in contributing to the development of the global obesity epidemic, indicating a link between PA and improved body composition [22]. However, the impact of PA on obesity may be in part determined by the genetic constitution of an individual [23,24,25,26]. Scientific evidence supports an interaction between PA and genetic background on obesity disposition, including evidence on the attenuating effect of PA on *TMEM18* obesity predisposition. Yet, specific genetic polymorphisms with a sensitivity to PA which are able to modify obesity risk remain widely unknown [27,28]. The biological mechanisms that underlie the attenuation of *TMEM18*’s effect in physically active individuals, and whether the interaction is due to PA or due to other environmental exposures remain unclear [27,28,29,30]. In this research, we further support the critical importance of PA as a weight loss-promoting behavior, as well as a prevention strategy of weight gain with an adequate regime that needs to be incorporated into clinical interventions for weight control with special attention given to *TMEM18* variant carriers.

We showed that both SSBs and flavored water consumption significantly interact with *TMEM18* rs939583 to elevate obesity risk. SSBs are defined as pre-packaged non-alcoholic water-based beverages, such as carbonated and energy drinks, which contribute free excessive and empty calories into the diet. The World Health Organization guidelines state that SSB consumption increases the overall energy intake and may lead to obesity and weight gain and elevate the risk of non-communicable diseases [31]. Initial studies have shown that the intake of SSBs interact with genetic predisposition factors, where a higher obesity risk was shown in people who were genetically predisposed to obesity with a greater intake of SSBs [32,33]. Indeed, our results elucidate the importance of the reduction or elimination of SSBs, including flavored water which contains added sugars, particularly in people with *TMEM18* SNPs predisposing obesity. Flavored water usually consists of water with liquid fructose, flavoring and aroma extracts, and other additives, containing 75 kcal and 5 tsp of added sugar in each bottle (500 mL). We choose to study the association of obesity with flavored water, as flavored water is a relatively new concept of sugary drinks category. Flavored water is marked by companies and adopted by consumers as a substitute for drinking water, although it contains empty calories. Therefore, we identified the importance of analyzing this category as an independent entity. One possible explanation for the interaction between *TMEM18* and SSBs and flavored water may be due to *TMEM18* expression in the hypothalamus area. The hypothalamus is heavily involved in appetite, food intake, and energy homeostasis via hormonal, and neural and food signals [8,34,35].

Our study demonstrated that weekly consumption of 1–3 glasses of wine reduced obesity risk by 35% (OR = 0.65 95% CI 0.52–0.81; *p* < 0.001) for *TMEM18* rs939583 variant homozygotes. American dietary guidelines recommend limiting alcohol consumption to a maximum of one glass per day for women and two glasses for men, where one glass of wine equals 150 milliliters [36]. These recommendations agree with one to three glasses consumed by our sample participants. Reports on the link between alcohol and obesity have shown to have either beneficial or adverse effects. Specifically, wine consumption was reported to be associated with anti-obesity properties [37]. Studies regarding the association between alcohol intake and genetic adiposity predisposition are scarce and inconsistent [38,39,40,41,42]. A possible mechanistic explanation for wine’s protective effect against obesity can be attributed to resveratrol. Resveratrol, a type of natural phenol found in food, including the skin of grapes, acts as an antioxidant with health-promoting properties, including BMI reduction [43,44]. Generally, studies investigating the interaction between variants predisposing obesity and wine consumption are lacking. To the best of our knowledge, this research is first to support the interaction between consuming wine and common *TMEM18* SNPs linked to obesity, indicating the need for further investigation and the replication of our results.

Our study has several limitations, including: the self-reported questionnaires and anthropometric indices, which may be inaccurate to a certain extent and may hinge on the participant’s mental state compared to measurements taken by a professional. Additionally, because of the cross-sectional nature of this study, findings are based on association rather than causation. However, the study’s strengths include; a large sample size, contributing to a greater precision of results. Moreover, we included the important analysis of the interaction of several common *TMEM18* genetic variations with lifestyle habits, as another step towards personalized nutritional guidelines. Furthermore, this is fundamental nutrigenetic evidence regarding *TMEM18* gene’s relation to obesity in the Israeli population.

## 5. Conclusions

Common *TMEM18* gene SNPs rs939583 and rs1879523 are significantly associated with obesity risk. Carriers of common *TMEM18* SNPs linked to obesity can modify their lifestyle behaviors that interact with their genetic predisposition, in particular, measurable PA and moderate wine consumption. For homozygote carriers of *TMEM18* rs939583 it is especially important to avoid the consumption of SSBs and flavored water. Our results add an additional layer for the implementation of personalized nutrition protocols for those who are predisposed to obesity.

## Figures and Tables

**Table 1:** Descriptive characteristics of study population.

	All Population n = 3089	Obese Subjects (BMI ≥ 30) n = 1743	Non-Obese Subjects (BMI < 30) n = 1346	*p* Value
Gender (women, %)	2136 (69.1%)	1155 (66.3%)	981 (72.88%)	**<0.001**
Age (mean ± SD, min, max)	55.21 ± 14.31 (18, 89)	55.49 ± 14.52 (18, 88)	54.85 ± 14.04 (18, 89)	0.11
Weight (mean ± SD, min, max)	87.86 ± 19.18 (40, 185)	98.69 ± 17.08 (64, 185)	73.82 ± 10.87 (40, 110)	**<0.0001**
Height (mean ± SD, min, max)	166.78 ± 8.89 (138, 198)	167.02 ± 9.18 (143, 198)	166.46 ± 8.5 (138, 195)	0.06
BMI (mean ± SD, min, max)	(31.49 ± 6.24) 15.24, 62	35.32 ± 5.51 (15.24, 29.98)	26.55 ± 2.55 (30, 62)	**<0.0001**
Physically active (n, %)	1621 (52.5%)	779 (44.7%)	842 (62.6%)	**<0.001**
Smoking (n, %)	310 (10%)	162 (9.3%)	148 (11.0%)	0.13
SSB Consumers (n, %)	364 (11.8%)	241 (13.8%)	123 (9.1%)	**<0.001**
Wine Consumers (n, %)	732 (23.7%)	364 (20.9%)	368 (27.3%)	**<0.001**
Flavored water Consumers (n, %)	209 (6.8%)	134 (7.7)	75 (5.6%)	**0.02**

Values in bold indicate significance of a *p* value < 0.05; SD: standard deviation; Min: minimum; max: maximum.

**Table 2:** TMEM18 SNP genotype frequency and obesity risk.

		Genotype Frequency (%)				*p* Value OR ± 95%(CI)				
SNP	Allele		Overall Population	Obese (n = 1743)	Non-Obese (n = 1346)	Dominant Model	Recessive Model	Additive Model	Codominant Model	Allelic Model
rs939583 (n = 3089)	C > T	CC TC TT	168 (5.4%) 1061 (34.3%) 1860 (60.2%)	85 (4.9%) 554 (31.8%) 1104 (63.3%)	83 (6.2%) 507 (37.7%) 756 (56.1%)	0.12 1.28 (0.94–1.75)	**<0.001** **1.35 (1.17–1.57)**	**<0.001** **1.27 (1.13–1.43)**	**0.03** **1.43 (1.04–1.96)**	**0.00004** **1.27 (1.27–1.43)**
rs18719523 (n = 3089)	A > T	AA AT TT	1458 (47.2%) 1341 (43.4%) 290 (9.4%)	791 (45.4%) 757 (43.4%) 195 (11.2%)	667 (49.6%) 584 (43.4%) 95 (7.1%)	**<0.02** **1.18 (1.03–1.36)**	**<0.001** **1.66 (1.29–2.15)**	**<0.001** **1.22 (1.09–1.36)**	**<0.001** **1.73 (1.33–2.26)**	**0.0002** **1.22 (1.09–1.36)**

Logistic regression adjusted for age, gender and T2DM. OR: odds ratio; CI: confidence interval. Bold values indicate significance of a *p* value < 0.05.

**Table 3:** Interaction between TMEM18 rs939583 and rs1879523 SNPs and actionable lifestyle factors.

SNP	Actionable Factors	β	OR ± CI	*p* Value
rs939583	PA	−0.3	0.74 (0.68–0.8)	**<0.001**
	SSBs	0.270	1.31 (1.14–1.51)	**<0.001**
	Flavored water	0.298	1.35 (1.13–1.62)	**0.001**
	Wine	−0.198	0.82 (0.74–0.9)	**<0.001**
rs1879523	PA	−0.223	0.8 (0.7–0.9)	**<0.001**
	SSBs	0.446	1.66 (1.26–2.26)	**<0.001**
	Flavored water	0.499	1.65 (1.17–2.33)	**0.005**
	Wine	−0.219	0.8 (0.67–0.96)	**0.02**

Logistic regression adjusted for age, gender and T2DM. OR: odds ratio; CI: confidence interval. Bold values indicate significance of a *p* value < 0.05.

**Table 4:** PA association with rs939583 TT alleles carriers and BMI.

PA Duration (Minutes)	SNP	BMI (Mean ±SD)	*p* Value * Ref: Inactive	*p* Value * Ref: Previous Duration Group
Not active (n = 1542)	rs939583	32.98 0.2		
30 min (n = 407)		32.27 0.67	**<0.005**	**0.005**
60 min (n = 552)		31.16 0.3	**<0.001**	**0.232**
90 min (n = 238)		29.91 0.4	**<0.001**	**0.01**
120 min (n = 171)		29.49 0.43	**<0.001**	**0.57**
Above 120 min (n = 62)		28.01 0.64	**<0.001**	**0.9**

* *p* value for the duration of activity groups compared to non-active.

**Table 5:** Wine and SSBs consumption on TMEM18 SNPs obesity risk.

		Wine Consuming				SSBs Consuming		
		β	OR ± CI	*p* Value		β	OR ± CI	*p* Value
rs939583	CC (n = 168)	−0.087	0.92 (0.46–1.82)	0.8	CC (n = 169)	0.778	2.18 (0.85–5.56)	0.1
	CT (n = 1061)	−0.508	0.6 (0.45–0.8)	**<0.001**	CT (n = 1062)	0.576	1.78 (1.2–2.63)	**0.004**
	TT (n = 1860)	−0.427	0.65 (0.52–0.81)	**<0.001**	TT (n = 1860)	0.391	1.47 (1.08–2.02)	**0.01**

Logistic regression adjusted for age, gender and T2DM. Bold values indicate significance of a *p*-value < 0.05.

**Table 6:** Flavored water consumption on TMEM18 rs939583 SNPs obesity risk.

		β	OR ± CI	*p* Value
rs939583	CC (n = 111)	−0.497	0.6 (0.14–2.67)	0.51
	TC (n = 688)	0.263	1.3 (0.72–2.36)	0.39
	TT (n = 1202)	0.761	2.14 (1.3–3.54)	**0.003**
	TT vs. CC + TC (n = 2138)	0.99	2.69 (1.28–3.65)	**0.009**
	TT vs. CC (n = 1313)	1.8	6.14 (1.4–27.1)	**0.017**

Logistic regression adjusted for age, gender and T2DM. Bold values indicate significance of a *p* value < 0.05.

## Data Availability

Not applicable.

## Supplementary Materials

The following supporting information can be downloaded at: https://www.mdpi.com/article/10.3390/nu15020266/s1, Table S1: Hardy–Weinberg equilibrium for the studied TMEM18 SNPs.
