# Supplementary material for: Drinking Habits and Physical Activity Interact and Attenuate Obesity Predisposition of TMEM18 Polymorphisms Carriers
Source: Nutrients. 2023 Jan 4;15(2):266. doi: 10.3390/nu15020266 (PMC9860767; doi:10.3390/nu15020266)
Supplement: Supplementary file 1 [file nutrients-15-00266-s001.zip › nutrients-2128770-supplementary.pdf]

Table S1. Hardy-Weinberg equilibrium for TMEM18 studied SNPs

SNP	Allele		Genotype frequency	MAF (%)	χ^2	*p*-Value
rs939583 (n=3091)	C>T	CC	169 (5.5%)	77.35	0.179	0.915
		TC	1062 (34.4%)			
		TT	1860 (60.2%)			
rs1879523 (n=3091)	A>T	AA	1460 (47.2%)	31.1	0.042	0.979
		AT	1341 (43.4%)			
		TT	290 (9.4%)			
rs2867125 (n=3090)	T>C	TT	168 (5.4%)	77.35	0.007	0.996
		CT	1064 (34.4%)			
		CC	1858 (60.1%)			
rs2903492 (n=3090)	G>A	GG	167 (5.4%)	77.24	0.006	0.997
		AG	1068 (34.6%)			
		AA	1855 (60.0%)			
rs4854344 (n=3085)	G>T	GG	175 (5.7%)	76.5	0.035	0.983
		GT	1100 (35.6%)			
		TT	1810 (58.6%)			
rs6548238 (n=3091)	T>C	TT	170 (5.5%)	77.11	0.109	0.947
		CT	1075 (34.8%)			
		CC	1846 (59.7%)			
rs10189761 (n=3082)	T>A	TT	177 (5.6%)	76.56	0.072	0.965
		AT	1091 (35.3%)			
		AA	1814 (58.7%)			
rs13021737 (n=3090)	A>G	AA	170 (5.5%)	77.06	0.107	0.948
		AG	1074 (34.7%)			
		GG	1845 (59.7%)			
rs7561317 (n=3091)	A>G	AA	175 (5.5%)	76.56	0.041	0.980
		AG	1099 (35.6%)			
		GG	1817 (58.8%)			

SNP: single nucleotide polymorphism; MAF: minor allele frequency; n: number of sample
